# Intergenerational effects of ocean temperature variation: Early life benefits are short-lived in threespine stickleback

**DOI:** 10.1371/journal.pone.0307030

**Published:** 2024-08-02

**Authors:** Helen Clare Spence-Jones, Carla M. Pein, Lisa N. S. Shama

**Affiliations:** Coastal Ecology Section, Alfred Wegener Institute Helmholtz Centre for Polar and Marine Research, List, Germany; University of Connecticut, UNITED STATES OF AMERICA

## Abstract

Current climate change models predict an increase in temperature variability and extreme events such as heatwaves, and organisms need to cope with consequent changes to environmental variation. Non-genetic inheritance mechanisms can enable parental generations to prime their offspring’s abilities to acclimate to environmental change–but they may also be deleterious. When parents are exposed to predictable environments, intergenerational plasticity can lead to better offspring trait performance in matching environments. Alternatively, parents exposed to variable or unpredictable environments may use plastic bet-hedging strategies to adjust the phenotypic variance among offspring. Here, we used a model species, the threespine stickleback (*Gasterosteus aculeatus*), to test whether putatively adaptive intergenerational effects can occur in response to shifts in environmental variation as well as to shifts in environmental mean, and whether parents employ plastic bet-hedging strategies in response to increasing environmental variation. We used a full-factorial, split-clutch experiment with parents and offspring exposed to three temperature regimes: constant, natural variation, and increased variation. We show that within-generation exposure to increased temperature variation reduces growth of offspring, but having parents that were exposed to natural temperature variation during gametogenesis may offset some early-life negative growth effects. However, these mitigating intergenerational effects do not appear to persist later in life. We found no indication that stickleback mothers plastically altered offspring phenotypic variance (egg size or clutch size) in response to temperature variation. However, lower inter-individual variance of juvenile fish morphology in offspring of increased variation parents may imply the presence of conservative bet-hedging strategies in natural populations. Overall, in our experiment, parental exposure to temperature variation had limited effects on offspring fitness-related traits. Natural levels of environmental variation promoted a potentially adaptive intergenerational response in early life development, but under more challenging conditions associated with increased environmental variation, the effect was lost.

## Introduction

Organisms in the wild are faced with constantly changing environments and frequently must alter their phenotypes to maintain fitness. Even when environmental change is not in itself stressful to an organism, it may become so if it occurs at a magnitude or frequency that exceeds the organism’s capacity to acclimate via phenotypic plasticity [1]. This is of particular concern given present-day predictions of increasing climate variability and extreme events such as heatwaves. The current rate of environmental change is already too rapid for genetic adaptation of some species [2], and is leading to increased frequency and intensity of environmental fluctuations in addition to directional changes to environmental mean [3–7].

To respond adaptively to environmental change, organisms must obtain information about the state of their environment and/or predict future conditions. Non-genetic inheritance mechanisms allow for information about recent ancestral environmental conditions to be passed from parent to offspring generations [8]. If environmental cues are reliable, parental (or grandparental etc.) information may prime offspring to perform better in matching future conditions [9]. This intergenerational plasticity may allow for potentially adaptive change in offspring right from the start of development [10]. However, such inheritance is not always adaptive. For example, offspring may directly inherit maladaptive factors, or mismatch between parental ‘priming’ and the actual environment experienced by offspring may result in maladaptive trait development [11]. The optimal strategy for a parent to maximize its reproductive fitness will depend on the predictability of environmental change [12, 13], with intergenerational plasticity favored in predictable conditions and bet-hedging strategies favored in unpredictable conditions. Bet-hedging is typically split into two major strategies: diversified and conservative. With diversified bet-hedging, within-clutch phenotypic variation is increased to raise the probability of high fitness for at least some individuals. By contrast, conservative bet-hedging is a strategy of reduced variation among offspring, for example ensuring all offspring have a low-risk, generalist phenotype [14, 15]. Such strategies can vary between species or populations [16], and bet-hedging itself may be a phenotypically plastic trait in the sense that parents may employ different bet-hedging strategies under different environmental conditions [17]. Importantly, intergenerational plasticity and bet-hedging strategies are not mutually exclusive [18–21].

Mechanisms governing such across-generation dynamics and the circumstances in which they are expected to occur, either adaptively or maladaptively, are not yet fully understood [22–24]. One aspect of this is whether adaptive intergenerational inheritance also can occur in response to environmental variation, as has been repeatedly shown in response to shifts in environmental mean [9]. Here, we use threespine stickleback (*Gasterosteus aculeatus*) as a model species to investigate these dynamics in response to ocean temperature variation, which is predicted to increase alongside climate change-induced ocean warming [4, 25]. While (putatively adaptive) intergenerational effects in response to mean temperature change in threespine stickleback have been extensively studied [26–30], responses to temperature variation both within and across generations are less well known. For instance, stickleback showed some evidence for plastic bet hedging of offspring size (higher phenotypic variance) in response to weekly switches between two temperatures [31], whereas no plastic bet hedging occurred when parents experienced stochastically fluctuating temperatures. Moreover, mean offspring size was reduced with diversified bet hedging, but largest when offspring were reared in stochastically fluctuating temperatures, regardless of parental acclimation environment [32].

Threespine stickleback, like most fish, are ectothermic and can respond to temperature change using a number of mechanisms. These include behavioral strategies to navigate towards optimal temperatures [33], and adjustments to their metabolic machinery to acclimate to different temperatures e.g. [34, 35]. Even when temperatures do not approach lethal limits, acclimation through these processes may require significant resource allocation, in addition to direct effects of temperature on metabolic rates. For example, Guderley, Leroy and Gagné [36] found that stickleback growth rates remained constant between 8°C and 23°C despite a doubling in feeding rate, implying considerable resource allocation to processes other than growth at higher mean temperatures. Temperature variation may exacerbate this by constantly forcing individuals to adjust their physiology to perform optimally, or may result in individuals reaching their adjustment limits, necessitating tolerance of transient episodes of sub-optimal metabolism. By contrast, temperature variation may be beneficial for ectotherm growth if they are able to seek out optimal temperatures for different developmental processes. For instance, Atlantic salmon (*Salmo salar*) kept under widely ranging temperatures during early post-hatch development showed improved survival and growth [37]. Thermal hardening, the physiological phenomenon whereby exposure to temperature variation–particularly transient exposure to sub-lethal high temperatures–can expand an individual’s thermal tolerance range and/or fitness at the limits of this range [38–40]. The capacity for thermal hardening can vary within and between species, and potentially allows organisms to maintain fitness despite temperature variation which would otherwise disrupt metabolic processes.

Here, we conducted an across-generation laboratory experiment using a full-factorial, split-clutch experimental design to assess within-generation and intergenerational responses of stickleback to a constant temperature, natural temperature variation, and increased temperature variation. We predicted that temperature variation beyond the natural range would be stressful for individuals and have negative impacts on fitness-relevant traits i.e. reproductive output, growth and/or survival. If adaptive intergenerational plasticity occurs, offspring grown in the same conditions their parents experienced (matching parent-offspring environments) were predicted to show improved trait performance relative to offspring of parents whose environmental experience did not match. If, however, plastic parental bet-hedging occurs in response to temperature variation, offspring of parents exposed to temperature variation were expected to show higher within-clutch phenotypic variance relative to offspring of parents in constant temperatures.

## Materials and methods

This study was conducted at the Wadden Sea Station Sylt [41] in accordance with German Animal Welfare Legislation and approved with written consent by the Animal Protection Commission (Tierschutz Kommission) of the Ministry for Agriculture, Rural areas, Europe and Consumer protection *(*Ministerium für Landwirtschaft, ländliche Räume, Europa und Verbraucherschutz: permit # V244-17922/2018 (38-4/18)).

Three temperature treatments were used in the experiment (S1 Fig): control (‘CON’, constant 18°C), which reflects the long-term mean summer temperature in the Sylt-Rømø Bight (55.05°N, 8.41°E) between 2010 and 2020, natural variation (‘NAT’), which reflects the natural temperature variation for this location [7], and increased variation (‘INC’), modelled by applying 2.5x the daily average temperature anomaly from the monthly mean temperature. Both variation treatments were generated using daily averaged local sea surface temperatures between 2010 and 2020 [7]. These temperatures do not approach the lethal limit for this population, as stickleback can tolerate temperatures as low as 4°C and as high as 30°C [42, 43], and maximum temperatures used in this study are consistent with those recorded in the local area (http://www.cosyna.de). Water temperatures in each treatment were controlled using a flow-through seawater system fitted with header tanks containing heaters (T-computer and 2 TH-500 heaters; Aqua-Medic, Bissendorf, Germany), and monitored hourly using data loggers (HOBO Pendant Loggers, Onset, MA, USA). Water temperatures in the experiment were changed daily for variation treatments, with changes ranging between 0–2.4°C (mean 0.5°C) absolute change between days for natural variation and 0–3.2°C (mean 1.0°C) absolute change between days for increased variation (S1 Fig). Temperatures recorded by the data loggers showed that the overall mean temperature across the entire experimental period was 17.9 ± 0.30°C in the constant treatment, 18.1 ± 1.00°C in natural variation (range 15.6–21.4°C), and 18.5 ± 1.66°C (range 14.5–22.1°C) in increased variation (see S1 Fig and Discussion).

Wild F0 adults (n = 164) were caught in the Sylt-Rømø Bight by trawling in April 2021 and immediately transferred to the laboratory. Adults were gradually acclimated to laboratory temperatures (from 7°C to 15°C at +1°C per day) over 9 days. Adults were then randomly split among the three temperature treatments and gradually acclimated for a further two days to the starting experimental temperatures (from 15°C to 18°C). Adults were housed in 25L tanks on flow-through systems (filtered seawater; pH = 7.85±0.02, O_2_ = 99.2–100% saturation, salinity = 28.8±0.5ppt, flow rate = 0.15–0.4L/minute) with a 12:12 L:D cycle and no more than 20 individuals per aquarium. After experiment day 12, the light cycle was switched to 14:10 L:D to simulate summer day length conditions and encourage adults to enter breeding condition [44]. For breeding adults, there were no significant length differences among treatment groups for either sex (males 53.2 ± 3.7mm, Kruskal-Wallis χ^2^_(2)_ = 2.847, p = 0.241; females 56.8 ± 5.2mm, Kruskal-Wallis χ^2^_(2)_ = 1.521, p = 0.467). Adults were fed defrosted bloodworms once daily (~0.1g/fish) and kept under experimental conditions for a minimum of 40 days before breeding (range 40–100 days acclimation at point of breeding). Previous studies of this population have shown that 5–6 weeks acclimation time is enough to promote differences in egg size [32] as well as DNA methylation and transcriptomic profiles of gonads [45] among temperature treatments. Moreover, a reproductive conditioning phase of this time span is far longer than the time required to develop a clutch of eggs in stickleback, which may have an inter-clutch interval as short as 3 days [46].

F1 offspring were generated using *in vitro* fertilization (see [27]) from F0 adults (CON: n = 22 families from 11 females and 22 males; NAT: n = 14 families from 7 females and 14 males; INC: n = 11 families from 8 females and 11 males) in the time window between 40 and 100 days after the temperature treatments were started. Note: not all females in each treatment became gravid during the acclimation phase, resulting in different numbers of families per treatment. Although the rate of entering breeding condition could not be compared among treatments since the number of non-breeding females within each treatment was unknown, timing of breeding was not significantly different among parental treatments (Kruskal-Wallis χ^2^_(2)_ = 1.163, p = 0.559). Fertilization rates were generally low within this experiment (median 22.9% of eggs within a clutch, IQR 11.6–36.4%), but did not differ significantly among parental treatments (Kruskal-Wallis χ^2^_(2)_ = 1.892, p = 0.388). Fertilized egg clutches were immediately split among the three temperature treatments (CON, NAT, and INC) into 1L aerated beakers of microfiltered seawater, and fry were fed *Artemia nauplii* daily *ad libitum* throughout the experiment. Half the volume of water in the beakers was changed weekly. At 14 days post-hatch, beakers with 14 or more fry were split among replicate beakers. After 30 days, up to 14 fry per family were transferred to 2L tanks on the flow-through water systems (with parameters as specified above) and maintained until 90 days post-hatch.

### 1.1 Response traits

Prior to fertilization, the number of eggs in each clutch was counted and mean egg size (egg diameter in mm) was measured in a subsample of 30 eggs per clutch using microscope imaging (ZEISS Stemi 508 with Axiocam 105 color at 64x magnification, ZEN 3.0 image processing). Clutches were monitored and unfertilized or spoiled eggs removed. Fertilization rates were estimated by counting the number of eggs showing visible signs of development after two days, and hatching success was estimated as the percentage of fertilized eggs which hatched. Offspring growth was measured as standard length (mm) of all offspring at 30, 60, and 90 days post-hatch using microscope imaging (see above). Individuals were removed from tanks using a net, dried with a paper towel, and laid briefly on calibrated graph paper for photography at 10x magnification before being returned to their tanks. Offspring counts at these points were used for survival analyses. Morphometrics were assessed using 22 two-dimensional landmarks (a subset of those used by [47]; see Fig 2) identified on microscope photographs of left- and right-facing offspring at 90 days post-hatch.

### Statistical analyses

All statistical analyses were performed in R (v4.3.1, [48]) using the packages ‘nlme’ [49], ‘PMCMRplus’ [50], ‘lawstat’ [51], ‘ggsurvfit’ [52], ‘survival’ [53], ‘MASS’ [54], ‘geomorph’ [55, 56], and ‘dplyr’ [57]. Plots were created using the package ‘ggplot2’ [58]. Means are reported with standard deviations unless otherwise specified.

### Fecundity

Only clutches with >10% fertilization rates (CON n = 17, NAT n = 5, INC n = 11) were used for analyses so as to exclude underdeveloped clutches which may not be representative of natural reproductive attempts. Clutch size was compared among parental treatments using a linear mixed-effect model with parental treatment, maternal length, and their interaction as fixed factors, and maternal ID as a random factor. Egg sizes were compared among clutches that contained 60–100 eggs (CON n = 6, NAT n = 9, INC n = 8; 30 eggs measured per clutch), as these clutch sizes were represented across all treatments. Egg size was compared across treatments using a linear mixed-effect model with parental treatment, clutch size, and the interaction between the two as fixed factors, and clutch ID nested within maternal ID as a random factor. Due to sampling error, one natural variation clutch was removed from egg size analyses. Variation in egg size within clutches across treatments was compared using a Kruskal-Wallis test of the coefficient of variation in egg size for each clutch. Variation in egg size between clutches across treatments was compared using a Brown-Forsythe test (Levene test based on deviation from the median) on the average egg diameter for each clutch. The percentage of fertilized eggs that hatched per clutch was not normally distributed (Shapiro-Wilk W = 0.899, p<0.001), and was compared among treatment combinations (parent-offspring treatment) using a Kruskal-Wallis test.

### Growth

As growth was density-dependent (Table 1, S2 Fig), any tanks with fewer than 6 fish at each sampling point (30, 60, and 90 days post-hatch) were excluded from analyses. Density therefore ranged between 6–14 fish per tank (parent (P):offspring (O) treatment combinations: P_CON:O_CON n = 10 tanks, P_CON:O_NAT n = 9 tanks, P_CON:O_INC n = 10 tanks, P_NAT:O_CON n = 7 tanks, P_NAT:O_NAT n = 9 tanks, P_NAT:O_INC n = 7 tanks, P_INC:O_CON n = 2 tanks, P_INC:O_NAT n = 4 tanks, P_INC:O_INC n = 3 tanks). Differences in standard length of fish at each sampling point were tested using linear mixed-effects models with density (number of fish in the tank), parent and offspring temperature treatment, and their interaction as fixed effects, and clutch ID nested within maternal ID as a random effect. Inter-individual length variation within family-treatment combinations was calculated as the coefficient of variation in standard length at each sampling timepoint. Inter-individual length variation was then compared among treatment combinations (parent-offspring treatment) using a Kruskal-Wallis test for each sampling timepoint.

**Table 1:** Results of linear mixed-models of standard length of offspring at each sampling point (30, 60 and 90 days post-hatch) depending on parent and offspring temperature treatments (constant, natural variation and increased variation).

	30-day Length		60-day Length		90-day Length	
	**Value ± Standard Error (mm)**	**p-value**	**Value ± Standard Error (mm)**	**p-value**	**Value ± Standard Error (mm)**	**p-value**
**Parent/Offspring Treatment—Constant (Intercept)**	**17.522±0.454**	**<0.001**	**23.399±0.648**	**<0.001**	**29.260±0.670**	**<0.001**
**Density**	**-0.219±0.038**	**<0.001**	**-0.460±0.050**	**<0.001**	**-0.693±0.058**	**<0.001**
**Parent Treatment—Increased Variation (P-INC)**	-0.520±0.592	0.395	-1.231±0.762	0.107	0.560±0.902	0.546
**Parent Treatment—Natural Variation (P-NAT)**	-0.453±0.461	0.342	-0.357±0.774	0.645	-0.589±0.725	0.431
**Offspring Treatment—Increased Variation (O-INC)**	**-0.488±0.170**	**0.004**	**-0.556±0.206**	**0.007**	**-0.980±0.237**	**<0.001**
**Offspring Treatment—Natural Variation (O-NAT)**	0.324±0.190	0.089	-0.035±0.222	0.873	0.221±0.256	0.388
**P-INC:O-INC**	0.038±0.326	0.908	0.585±0.387	0.131	-0.189±0.451	0.676
**P-NAT:O-INC**	**0.718±0.260**	**0.006**	-0.061±0.330	0.854	0.101±0.366	0.784
**P-INC:O-NAT**	-0.132±0.333	0.692	0.318±0.388	0.413	-0.765±0.457	0.095
**P-NAT:O-NAT**	-0.385±0.268	0.152	-0.200±0.338	0.554	-0.170±0.368	0.644
**Clutch ID and Maternal ID (random effects)** Maternal ID st.dev, [nested] Clutch ID st dev, (residual st. dev)	0.0003, 1.027, (1.049)		0.946, 1.313, (1.232)		0.771, 1.140, (1.452)	

Clutch ID was included in the model as a random effect, nested within Maternal ID. Significant effects (p<0.05) are shaded and highlighted in bold. ‘P-’ and ‘O-’ refer to ‘parental treatment’ and ‘offspring treatment’ respectively, and treatments are abbreviated to ‘INC’ (increased temperature variation) and ‘VAR’ (natural temperature variation).

### Post-hatch survival

Post-hatch survival was assessed using offspring survival up to 90 days (measured at 30, 60, and 90 days) with a Cox proportional hazard model containing parental treatment, offspring treatment, and their interaction as factors.

### Morphometrics

As with the growth analyses, only individuals from tanks with at least 6 fish were used for morphometric analyses (P_CON:O_CON n = 120 individuals, P_CON:O_NAT n = 110, P_CON:O_INC n = 101, P_NAT:O_CON n = 75, P_NAT:O_NAT n = 92, P_NAT:O_INC n = 74, P_INC:O_CON n = 43, P_INC:O_NAT n = 43, P_INC:O_INC n = 43). Two individuals lacked an eye on one side, and one had two spines rather than three. Missing landmark positions for these individuals were extrapolated using thin-plate spline interpolation [59]. Generalized Procrustes Analysis with matching symmetry on 22 landmarks (Fig 2) on both left and right sides of each fish was used to obtain Procrustes-transformed landmarks representing the average shape and unsigned asymmetry index (a metric of fluctuating asymmetry, a proxy for developmental instability [60–62]) for each individual.

Morphological variation between individuals (controlling for allometry) was analyzed using a Procrustes MANOVA (1000 permutations; [63]) with individual length, parental treatment, offspring treatment, and the interaction between parental and offspring treatment as fixed factors. Principle Components Analysis was used to determine the major axes of morphological variation between individuals. A Linear Discriminant Analysis (Canonical Variates Analysis) was used to generate jack-knifed cross-validations of assignment accuracy between treatment combinations, as a metric of the reliability of morphological differences between groups [61]. Differences in inter-individual variation among treatment groups were assessed using analysis of Procrustes Variances (controlled for individual length and using group means) to test for pairwise differences between parent-offspring treatment groups with a randomized residual permutation test (1000 iterations; [64, 65]). Differences in within-individual morphological variation (left-right fluctuating asymmetry) among groups were assessed using Generalized Linear Modelling of individual unsigned asymmetry index, with individual length, parental treatment, offspring treatment, and the interaction between parental and offspring treatment as fixed factors.

## Results

### Fecundity

Overall mean clutch size in the experiment was 97.3±28 eggs, and was not significantly affected by parental treatment (F_(2,15)_ = 1.784, p = 0.202), maternal length (F_(1,15)_ = 2.902, p = 0.109) or the interaction between the two (F_(2,15)_ = 0.733, p = 0.497). Mean egg size was 1.6±0.08μm. Egg size was not significantly different among parental treatments (F_(2,9)_ = 0.739, p = 0.505), and was not affected by clutch size (F_(1,3)_ = 1.143, p = 0.363) or its interaction with parental treatment (F_(2,3)_ = 1.909, p = 0.292). Neither within-clutch egg size variation (Kruskal-Wallis χ^2^_(2)_ = 3.17, p = 0.205) nor inter-clutch egg size variation (Brown-Forsythe = 0.523, p = 0.597) were significantly different among parental treatments. Hatching success (percentage of fertilized eggs that hatched) was not significantly different among parent-offspring treatment groups (Kruskal-Wallis χ^2^_(8)_ = 6.912, p = 0.546).

### Offspring growth

**Fig 1:**
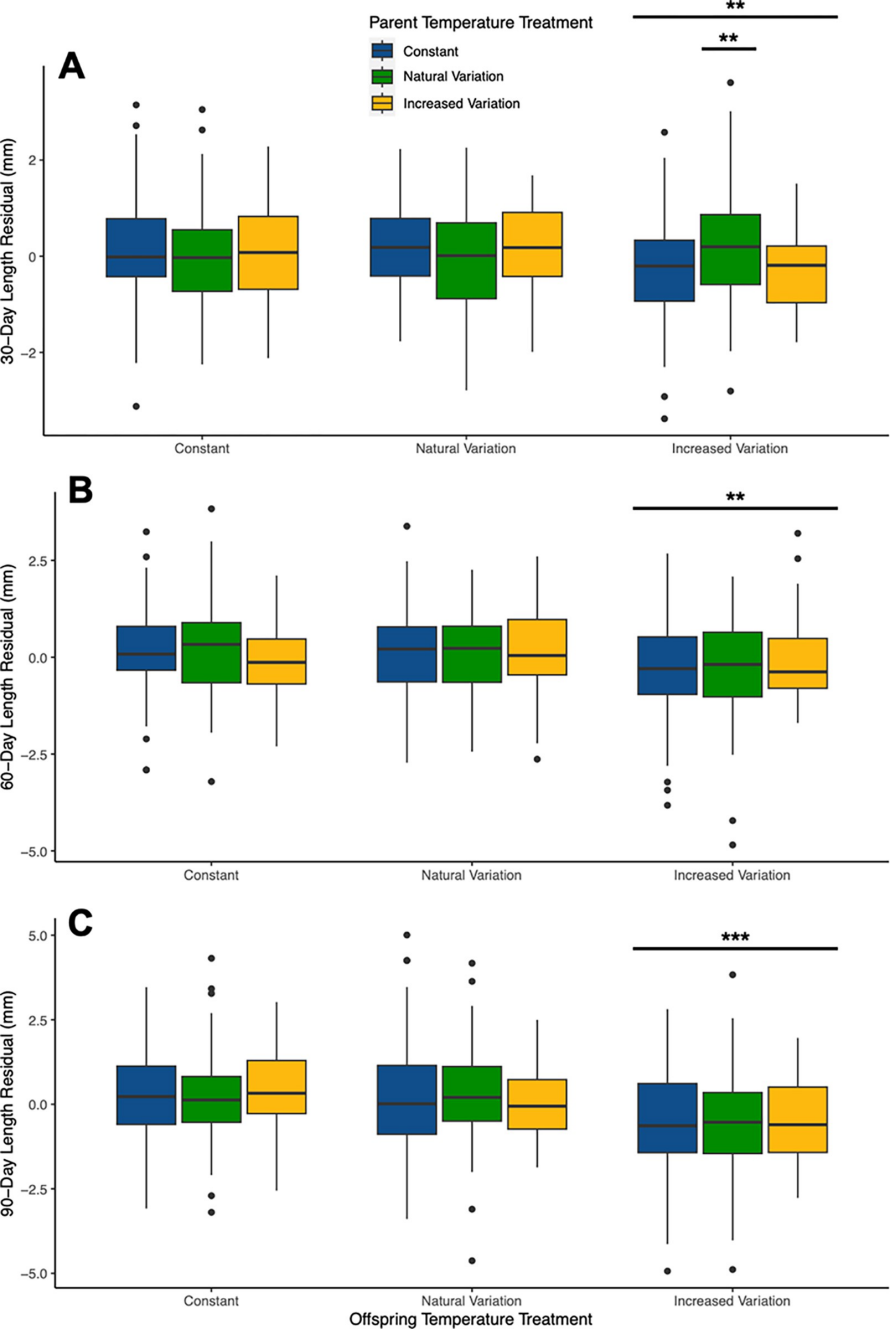
Tukey boxplots of density-corrected length of F1 generation stickleback offspring at (A) 30 days, (B) 60 days, and (C) 90 days for the nine parent-offspring temperature combinations (constant, natural variation, and increased variation for both parents and offspring). Length residuals corrected for the number of fish in the tank using a linear model are shown (see Methods). Bars indicate significant effects of O-INC throughout the experiment, and P-NAT:O-INC at 30 days (Table 1). Stars indicate significance at <0.001 (***) and <0.01 (**). Treatment medians are shown with upper and lower quartiles; whiskers extend to the largest value no further than 1.5*Inter-quartile range from upper/lower quartile, and points beyond this are displayed as outliers.

Throughout the experiment, offspring reared in the increased variation treatment (O_INC) were significantly smaller than their siblings in constant (O_CON) and natural temperature variation (O_NAT; as indicated in Table 1 and Fig 1). At 30 days, however, offspring reared in increased variation whose parents were acclimated to natural variation (P_NAT:O_INC) were significantly larger than their siblings in the other offspring treatments (Fig 1A). However, this beneficial intergenerational effect did not persist to 60 or 90 days (Table 1). Inter-individual variation in length was not significantly different among treatments at any sampling point during the study (30 days: Kruskal-Wallis χ^2^_(8)_ = 12.201, p = 0.142; 60 days: Kruskal-Wallis χ^2^_(8)_ = 3.872, p = 0.869; 90 days: Kruskal-Wallis χ^2^_(8)_ = 6.345, p = 0.609).

### Offspring survival

Survival to 90 days (91.8% across all fish) was not significantly affected by parental treatment, offspring treatment, or the interaction between the two (Cox Likelihood ratio test _(8)_ = 9.17, p = 0.328).

### Offspring morphometrics

#### Differences in morphology

**Fig 2:**
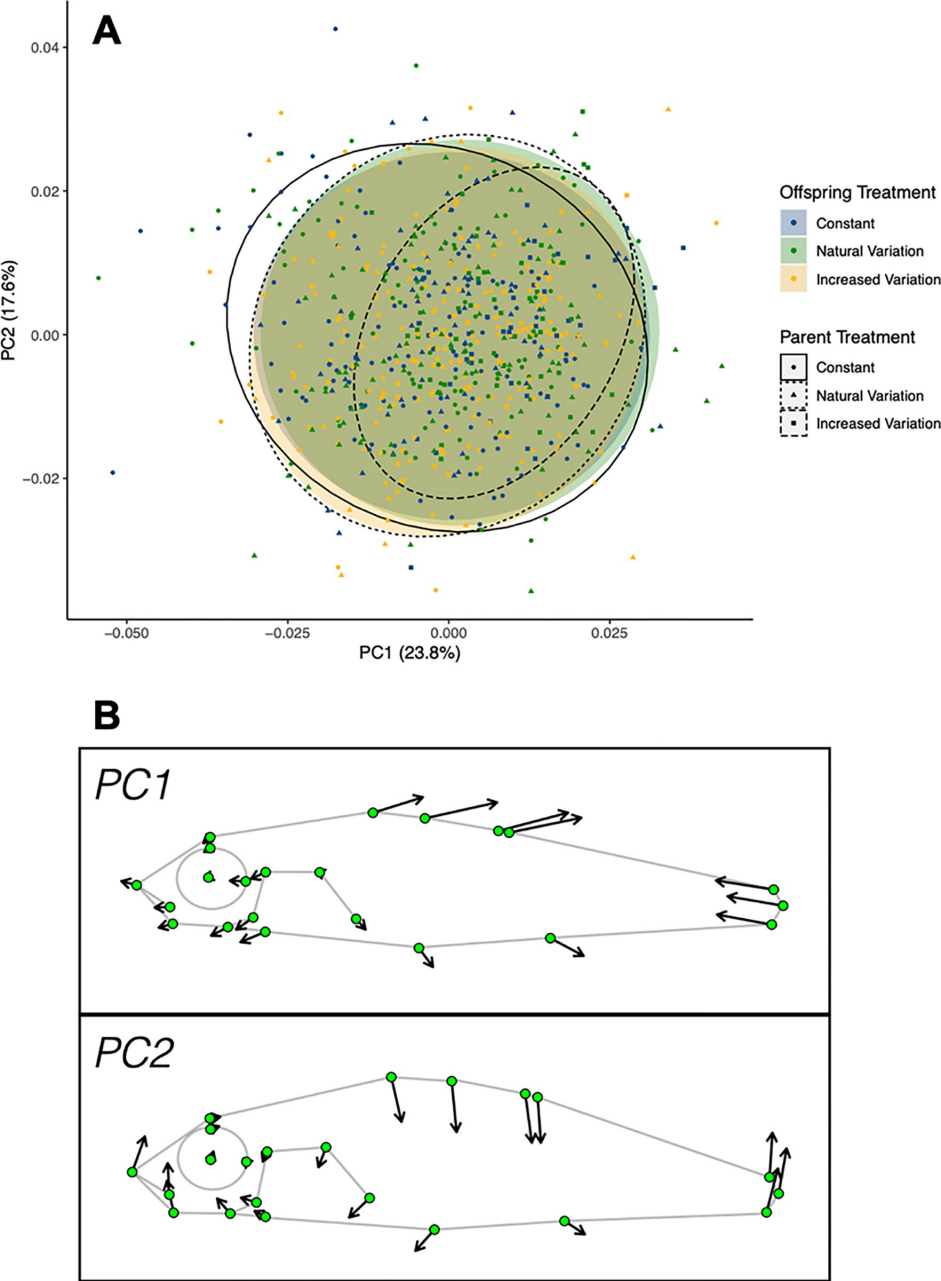
(A) PCA (Principal Component Analysis) of landmark coordinates revealed that the majority of stickleback morphological variation was not associated with either parental or offspring temperature treatment (points represent first and second PC scores of individuals; ellipses depict 95% confidence intervals). (B) Lollipop visualization of the morphological variation associated with the first two principal components (PCs) are shown, illustrating how shape changes along the PC with a scaling factor of 2. Arrows represent shape change as PC score increases, with a longer arrow representing more change associated with the PC. The majority of variation was in tail length (PC1) and body depth (PC2).

Fish within this experiment tended to show variation in body depth and relative tail length (Fig 2). There was a significant relationship between morphology and length (F_(1)_ = 37.653, R^2^ = 0.050, p<0.001). Additionally, there were small but significant differences in morphology between parental (F_(2)_ = 8.277, R^2^ = 0.022, p<0.001) and offspring (F_(2)_ = 2.440, R^2^ = 0.006, p<0.001) treatments, but no significant interaction effect (F_(4)_ = 1.281, p = 0.131; see S3 Fig for visualizations of average morphologies per treatment group). However, in both cases, low R^2^ values imply that the majority of morphological variation was not associated with experimental treatment (see Fig 2A). This was supported by a jack-knifed linear discriminant analysis, which demonstrated between 9 and 35% classification accuracy to (parent-offspring combination) treatment group, and an average of 27.5% correct classification.

#### Differences in morphological variation

**Fig 3:**
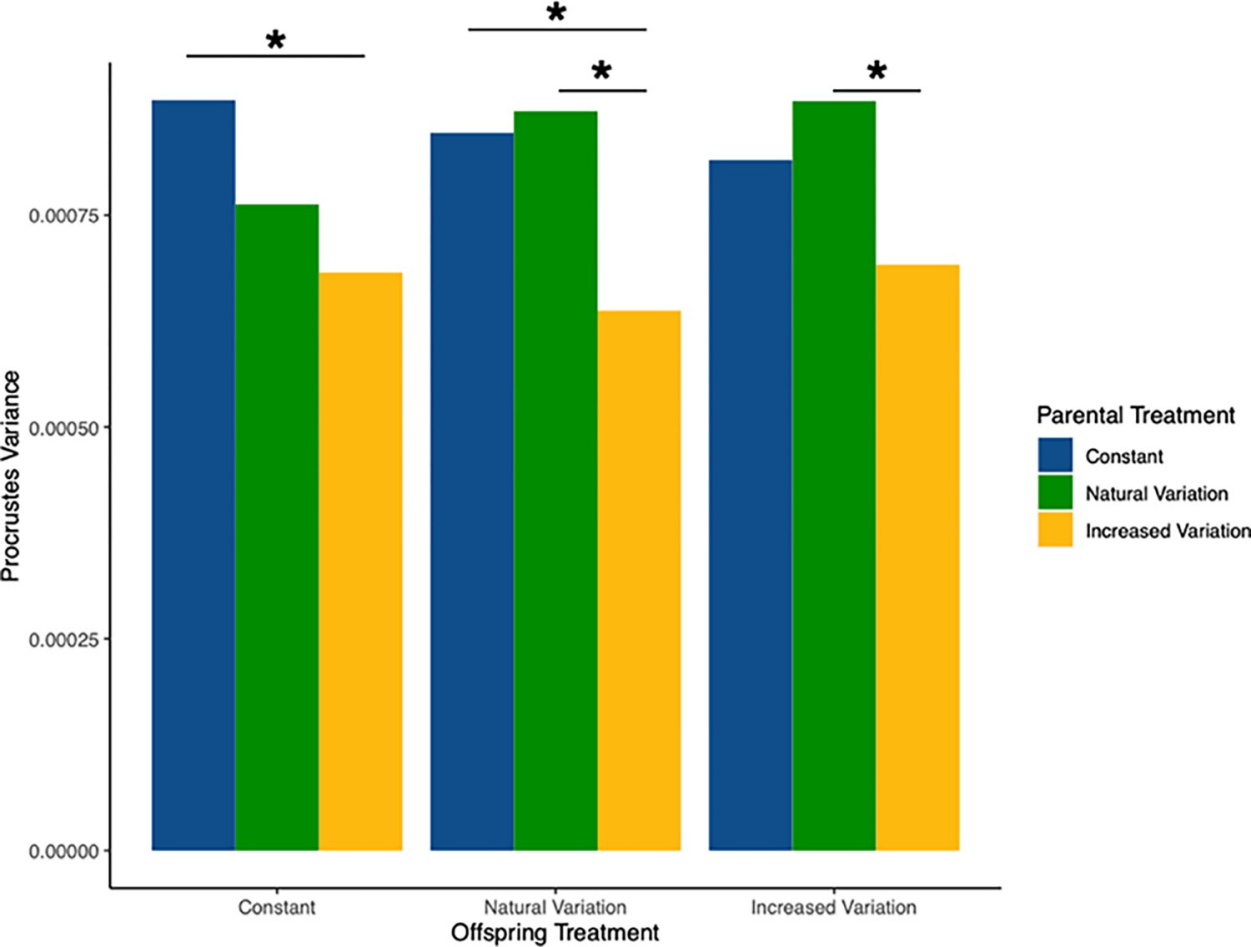
Within-group inter-individual morphological variation (Procrustes variance) for the nine parent-offspring temperature combinations (constant, natural variation, and increased variation for both parents and offspring), showing generally lower inter-individual morphological variation for offspring of parents in increased-variation. Starred bars indicate significant differences (p<0.05) within offspring treatment conditions (see Table 2).

Inter-individual morphological variation was, in general, significantly lower for individuals with increased-variation parents (P_INC) than those with constant temperature (P_CON) or natural variation (P_NAT) parents (Table 2; Fig 3). There were no significant differences in inter-individual morphological variation between offspring of parents in control vs natural variation treatments. Within each parental treatment group, there were no significant effects of offspring treatment on inter-individual morphological variation (Table 2). Fluctuating asymmetry (left-right morphological variation) in individuals was not significantly associated with length (F_(1)_ = 0.613, p = 0.434), offspring treatment (F_(2)_ = 0.129, p = 0.879), parental treatment (F_(2)_ = 0.844, p = 0.431) or the interaction between parent-offspring treatment groups (F_(4)_ = 1.326, p = 0.259).

**Table 2:** P-values from randomized residual permutation test (RRPP; 1000 iterations) for pairwise comparisons of inter-individual morphological variation (Procrustes Variances) for parent:offspring treatment combinations.

		Parent: Constant			Parent: Natural Variation			Parent: Increased Variation		
		Offspring: Constant	Offspring: Natural Variation	Offspring: Increased Variation	Offspring: Constant	Offspring: Natural Variation	Offspring: Increased Variation	Offspring: Constant	Offspring: Natural Variation	Offspring: Increased Variation
**Parent: Constant**	**Offspring: Constant**		0.510	0.247	0.057	0.841	0.988	0.008	0.003	0.015
	**Offspring: Natural Variation**	0.510		0.608	0.202	0.704	0.559	0.038	0.012	0.047
	**Offspring: Increased Variation**	0.247	0.608		0.446	0.375	0.328	0.096	0.030	0.135
**Parent: Natural Variation**	**Offspring: Constant**	0.057	0.202	0.446		0.110	0.099	0.334	0.149	0.399
	**Offspring: Natural Variation**	0.841	0.704	0.375	0.110		0.874	0.020	0.005	0.027
	**Offspring: Increased Variation**	0.988	0.559	0.328	0.099	0.874		0.020	0.003	0.029
**Parent: Increased Variation**	**Offspring: Constant**	0.008	0.038	0.096	0.334	0.020	0.020		0.637	0.926
	**Offspring: Natural Variation**	0.003	0.012	0.030	0.149	0.005	0.003	0.637		0.557
	**Offspring: Increased Variation**	0.015	0.047	0.135	0.399	0.027	0.029	0.926	0.557	

Significant p-values (p<0.05) are highlighted.

## Discussion

Our study shows, broadly, that while natural levels of temperature variation do not have negative effects on stickleback growth (under laboratory conditions), increased temperature variation 2.5x above this leads to lower growth of juvenile fish. However, negative effects of increased temperature variation may be mitigated in early life through intergenerational effects if parents experienced natural temperature variation themselves. By contrast, we did not find this mitigating intergenerational effect for offspring of parents which experienced increased temperature variation. Stickleback mothers did not appear to plastically alter offspring phenotypic variance in response to environmental variation in terms of egg size or clutch size bet-hedging, but reduced inter-individual morphological variance among offspring of increased variation parents may imply some degree of conservative bet-hedging.

### Bet-hedging under temperature variation

Although theory predicts that environmental variability/unpredictability should favor phenotypically plastic and/or bet-hedging responses in parents [18, 31, 66], we found no evidence that mothers plastically altered their overall or per-offspring investment in response to temperature variation. Mean egg size and clutch size were not different among treatments, and mothers showed no evidence of plastic bet-hedging strategies such as manipulation of within-clutch size variation [15] in response to temperature variation. This was also reflected in clutch survival, which was not significantly different among parental (or offspring) treatments. These findings are in contrast to a previous study in the same stickleback population [31] where mothers in variable environments (in that study, switching weekly between two temperatures) produced a greater range of offspring sizes than mothers in constant temperatures. However, the current study used unpredictable day-to-day temperature fluctuations that simulated the natural variation experienced by this population (based on 10 years of temperature data recorded at the study site [7]). It may be that in cases where plasticity of bet-hedging does occur, alternate strategies are employed by parents in response to differing predictabilities of temperature variation or patterns of environmental variation [12, 13, 67, 68], which could not be tested in our study as temperature variation treatments differed in the amplitude of fluctuations, but not in the predictability (temperatures were changed on the same day but to different extents). Our study does not preclude maternal manipulation of egg properties or quality, since mothers can alter biochemical aspects of egg content such as lipid or protein content without changes in egg size [69]. Investigating potential differences in egg content or (epi)genetic profiles (see [70]) in response to changes in both amplitude and predictability of temperature variation could provide valuable insight into mechanisms underlying plastic responses to future environmental uncertainty.

### Temperature variation effects on growth

Overall, we found negative within-generation plasticity of offspring size in response to the increased variation treatment, suggesting that 2.5x variation beyond the natural range was stressful for fish in terms of reduced growth. Across all sampling timepoints, offspring raised in increased variation were on average smaller than their siblings in constant temperature or natural temperature variation treatments. This occurred despite a slightly higher overall mean temperature in this treatment compared to the control (18.5°C vs 18°C; S1 Fig), which would be expected to result in increased rather than decreased growth [42, 71, 72]. Previous experiments in this population indicate that optimal growth in constant temperature conditions occurs somewhere between 18.5°C and 21°C [26, 27, 31, 32, 45, 73]. In this experiment, high temperatures (above 21°C) occasionally experienced in the increased variation treatment likely contributed to reduced overall offspring growth with little evidence for within-generation thermal hardening occuring [32]. Thermal hardening would be expected to manifest as consistent growth across offspring treatments, as transient exposure to high temperatures should result in increased tolerance to them [74]. Aspects such as the timing, magnitude and duration of temperature variation necessary to trigger phenotypic effects are yet to be investigated. Critical thermal limits are functions of both the intensity and duration of exposure, but implications for sub-lethal effects are not well known [75, 76].

Importantly, growth differences were not likely solely due to differences in mean temperature. Mean temperatures in the natural variation treatment were also higher than 18°C during some periods of the experiment, but we did not see a corresponding decrease in growth for those offspring. Moreover, offspring in natural temperature variation were significantly larger than offsping in increased variation, indicating that the amplitude of temperature variation also played a role in growth responses, with 2.5x beyond the natural range being stressful, whereas natural variation was optimal for growth. Interestingly, negative growth effects of increased variation were offset in early life for individuals whose parents experienced natural variation. It is notable that this beneficial intergenerational effect did not extend to offspring of parents who experienced increased variation. Thus, it may be that such (potentially) adaptive intergenerational plasticity is costly, and parents that experience a stressful environment (increased variation or transient temperature extremes) may be allocating substantial resources to other processes (e.g. maintaining metabolism [36]), and do not or cannot `prime´ offspring for future stressful environments [77–79].

Any benefits of parental experience on offspring growth in this system appear to be restricted to early-life development. Although maternal effects may last long-term [80] and our experiment did not test for parental effects beyond 90 days of offspring life, they are often considered to be most pronounced in early development when parental influence on offspring environment is strongest [81]. However, Moore *et al*. [24] suggest that maternal effects are most pronounced in juvenile stages rather than embroynic (or adult) stages, and that the relative strength of maternal effects at different life stages varies with trait type (e.g. physiology, behaviour, etc.). Stickleback juveniles (fry) disperse from nests at around 6-10mm [82], meaning that older fry may be more able to move (laterally or vertically) to thermoregulate by seeking preferred temperatures [33]. That the beneficial intergenerational effect of a natural variation parent for offspring in increased variation conditions did not persist to 60 or 90 days may also imply that the mechanisms governing (relatively) increased growth to 30 days are costly. Such individuals may have an early advantage for growth, but if the stressful conditions persist, they fall back alongside individuals who did not have the same initial boost. For instance, reduced resource allocation to reproductive output or egg quality by temperature-stressed parents [66] is compensated for by offspring at first [11], only to later have to pay back the costs of such compensatory growth [83], which may be challenging in prolonged stressful conditions. In this case, however, it would be expected that individuals in the most stressful combination of treatments–those with increased variation parents which were themselves in increased variation conditions–would show the lowest growth, which was not observed. It may also be that there is a window of salient temperature variation during the reproductive cycle that our experiment did not catch. For instance, although stickleback can ovulate and lay clutches within 3 days of each other [46], oogenesis occurs at the end of the preceding summer, and vitellogenesis (yolk deposition) may begin before the breeding season starts, in winter or early spring [84]. Long time periods before reproduction may not allow for accurate prediction of environmental conditions. For example, sea surface temperatures are only predictable for up to 9 days in the future for this population [31], although this refers to mean temperature rather than temperature variation. Beyond this, increasing difficulty of environmental prediction is likely to lessen the potential for adaptive intergenerational plasticity [22].

An alternate perspective is that the decreased growth found here under increased temperature variation may not be maladaptive. It is a common assumption that higher growth is associated with larger size, and hence, higher fitness within populations of this species (particularly for females, as larger females lay larger clutches [85]). In a recent stickleback study, individuals showing low growth as a result of temporary thermal stress then displayed catch-up growth during the breeding season, but incurred costs in terms of oxidative DNA damage, decreased fertility and reproductive output [86]. However, it is possible that temperature variation favours smaller individuals. Across ectotherm species, smaller organisms have higher short-term tolerance of thermal extremes (but lower long-term thermal tolerance) than larger organisms [76]. While increased (mean) temperatures are often associated with decreases in body size in ectotherms, the lower metabolic demands of a smaller body size may partially offset increased metabolic rate (and associated oxidative stress) in high temperatures [87]. Furthermore, smaller size may allow a greater proportion of energy allocation to fitness-related processes such as courtship and mating, as previously demonstrated in stickleback from this study population [30]. Still, within-species associations between temperature variation and fitness are as yet mostly unknown.

### Fish morphology in variable environments

Rearing temperature is known to have significant effects on plasticity of stickleback morphology. In this population, fish raised at 21°C (compared to 17°C) were characterized by reduced relative size of the ectocoracoid, operculum and pelvic girdle [73]. Also, heritable morphological differences between warm and cold populations in Iceland showed deeper mid-body and caudal regions, as well as steeper craniofacial profiles in warmer habitats [88]. While there were significant morphological differences between offspring from different treatment groups within this study (see Fig 2, S3 Fig), they were relatively minor in terms of the overall morphological variation present. Moreover, we did not detect significant interactive effects between parent and offspring temperature treatments.

Individual fluctuating (left-right) asymmetry was also not significantly affected by treatment, implying that while increased temperature variation temperature was sufficiently stressful to affect growth rates, it did not have a negative impact on developmental processes involved in gross morphology. Rather, inter-individual morphological variation was significantly lower in offspring from increased-variation parents, a pattern consistent with conservative bet-hedging. This is in contrast to results found by Magierecka et al. [89], who demonstrated in stickleback that stressful environmental unpredictability (in terms of light/dark periods, water turbulence, shelter availability, and chase and/or capture) for parents resulted in increased inter-individual behavioral variation within a clutch. Differences between these responses to variation may lie in the differing nature of the environmental cues, and in physiological versus behavioral responses. While increased behavioral flexibility and diversity may be appropriate for the stressful conditions described above, mothers in stressful temperature fluctuations may be engaging in conservative bet-hedging to produce a temperature-generalist phenotype with a baseline fitness across a wide range of temperature conditions. However, the adaptive significance of morphology with regards to temperature conditions within this species is still unclear [73, 88]. It is possible that different body morphologies favor different swimming styles or influence ability to occupy different water depths or current strengths [1, 90, 91].

## Conclusion

Mild parental stress in terms of temperature variation may confer beneficial intergenerational effects on individuals under stressful environmental variation in early life, but this does not appear to persist beyond early development. We found little evidence for plastic bet-hedging of offspring size in response to environmental variation, although offspring of increased variation parents did show reduced variation in body morphology, perhaps implying parentally-mediated conservative bet-hedging strategies. Understanding the mechanisms governing different forms of non-genetic inheritance in response to increasing environmental variation in terms of their impact on fitness-related traits may be fruitful for better predictive capacity of the circumstances under which they are expected to occur, their influence on intrapopulation phenotypic variance, and ultimately, the adaptive potential of populations to future climate uncertainty.

## Supporting information

S1 Fig Temperatures used for all treatments across the experimental period: dashed vertical lines indicate the start and end of the breeding period for adults. 14:10 light:dark photoperiod was started on day 12 and continued throughout the experiment for both adults and offspring.
(TIF)

S2 Fig Density-dependence of fish length for fry hatched during this experiment. Tanks with fewer than 6 fish were excluded from length analyses as the relationship between fish density in the tank and length became non-linear at low densities, particularly as fish grew older.
(TIF)

S3 Fig Wireplots showing shape deformation between the mean shape for each treatment group relative to the P_Con:O_Con control group. Note that all inter-group shape variation was relatively small within this experiment; plots are scaled by a factor of 6 to show differences.
(TIF)
