# Supplementary figures and images for: Intergenerational effects of ocean temperature variation: Early life benefits are short-lived in threespine stickleback
Source: PLoS One. 2024 Aug 2;19(8):e0307030. doi: 10.1371/journal.pone.0307030 (PMC11296643; doi:10.1371/journal.pone.0307030)

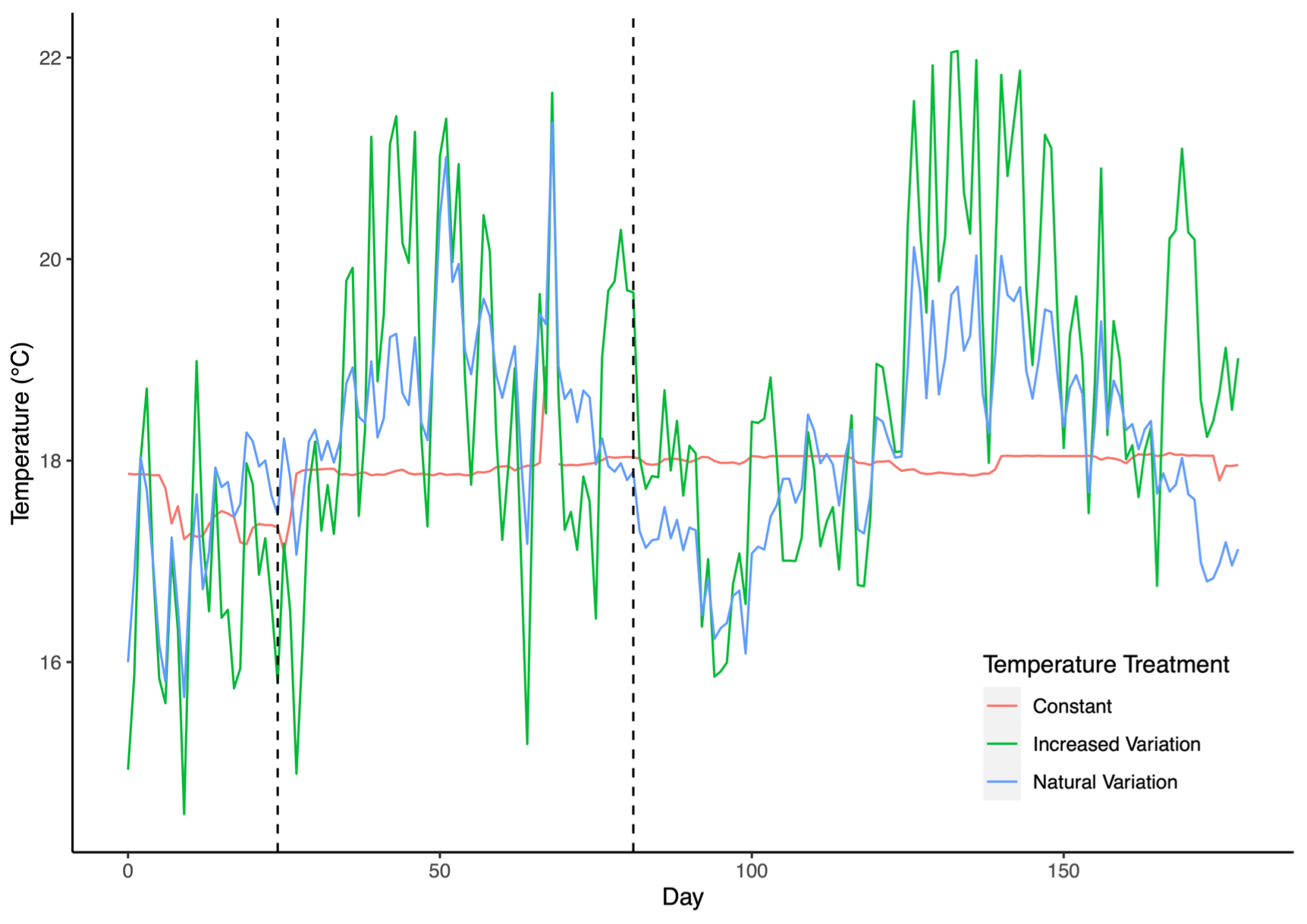

Supplement: S1 Fig — 14:10 light:dark photoperiod was started on day 12 and continued throughout the experiment for both adults and offspring. (TIF) [file pone.0307030.s001.tif]

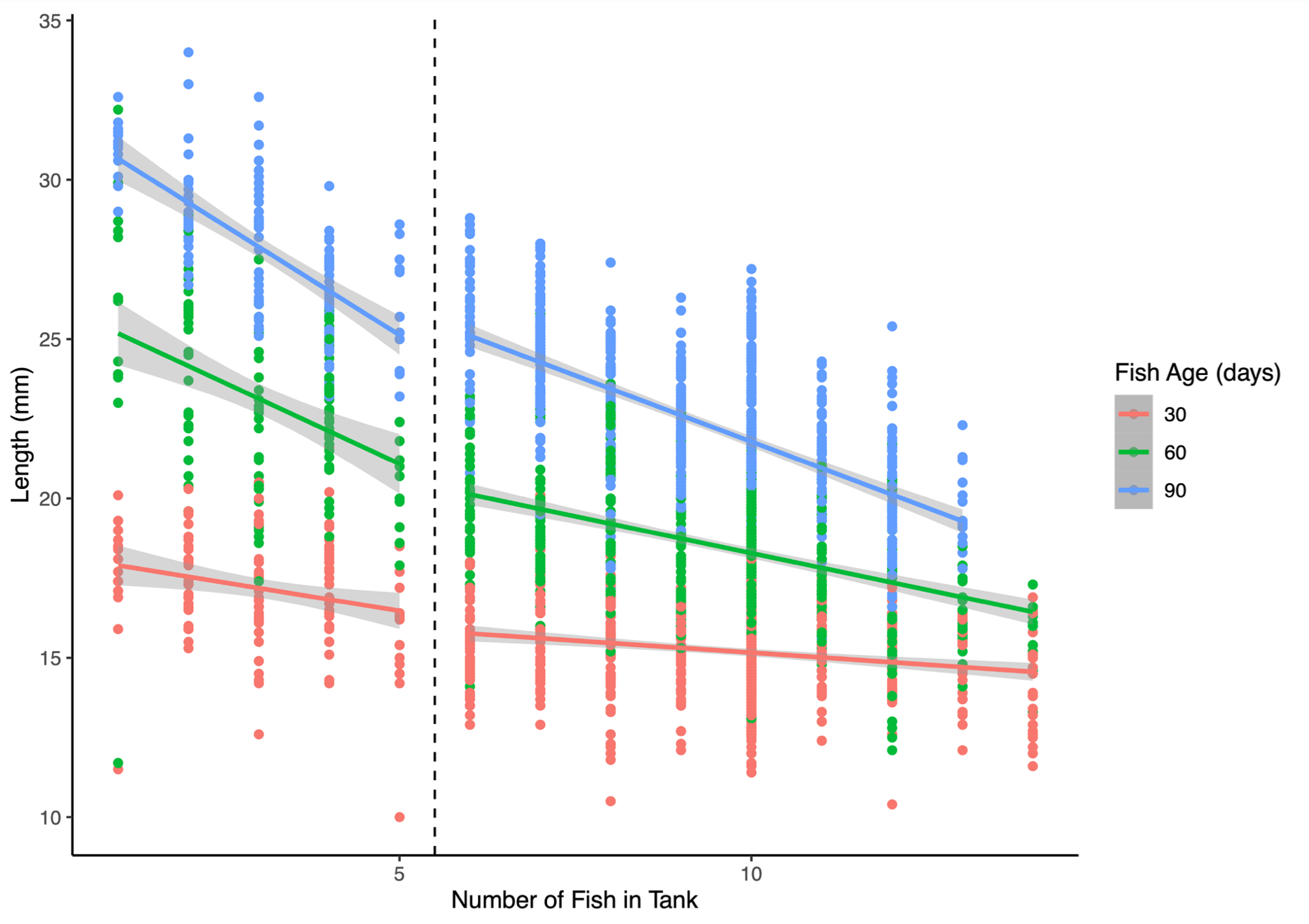

Supplement: S2 Fig — Tanks with fewer than 6 fish were excluded from length analyses as the relationship between fish density in the tank and length became non-linear at low densities, particularly as fish grew older. (TIF) [file pone.0307030.s002.tif]

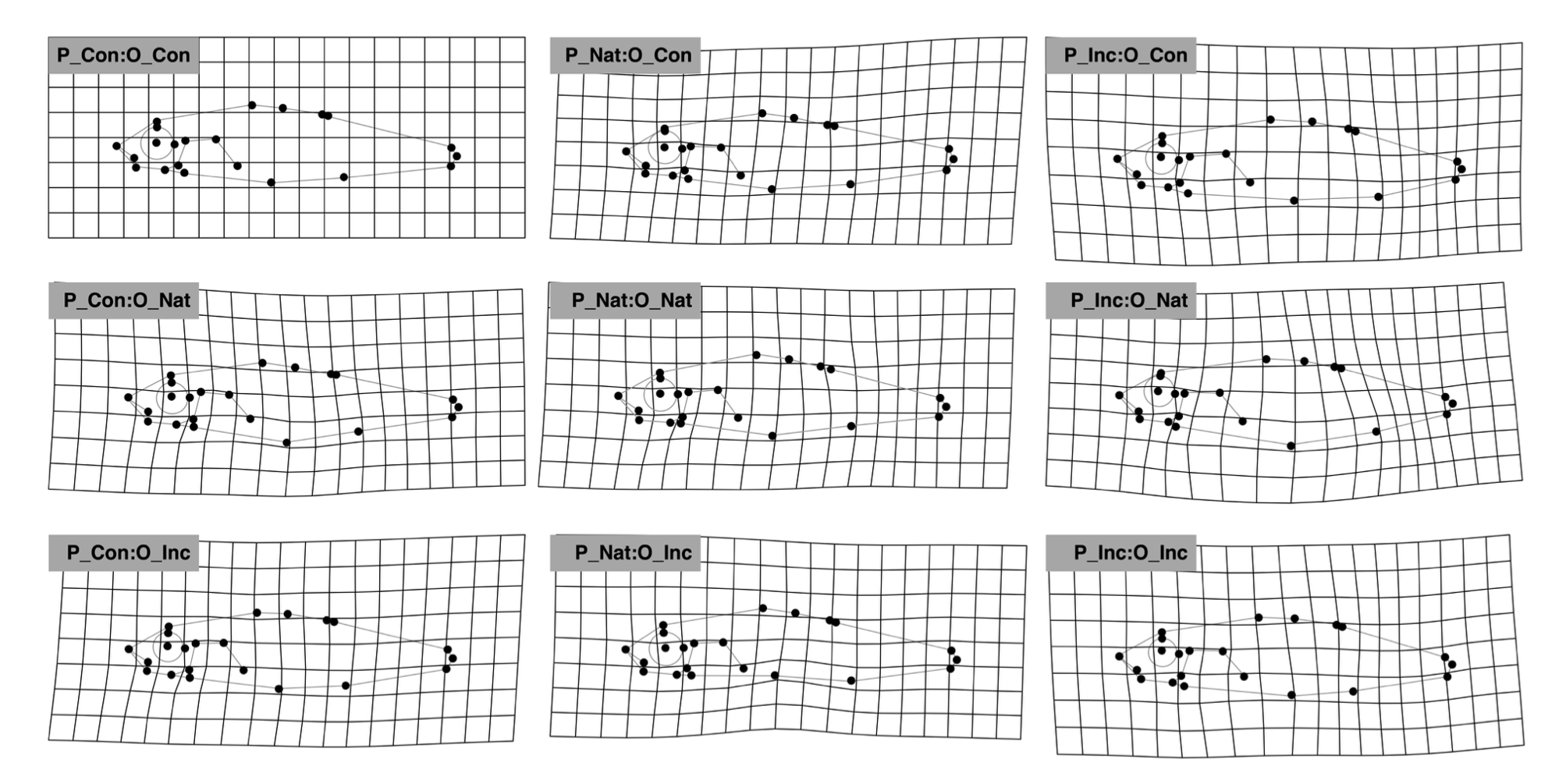

Supplement: S3 Fig — Note that all inter-group shape variation was relatively small within this experiment; plots are scaled by a factor of 6 to show differences. (TIF) [file pone.0307030.s003.tif]
